# Development and initial validation of the attitudes toward face mask use scale (ATFMUS)

**DOI:** 10.1016/j.heliyon.2022.e12349

**Published:** 2022-12-19

**Authors:** Anthony Muriithi Ireri, Cecilia Nyambura Mwangi, Vera Arhin, Martha Oigo, Stephen Mugo, Ruth Ncororo Munanu

**Affiliations:** a Department of Educational Psychology, Kenyatta University, Nairobi, Kenya; b College of Distance Education, University of Cape Coast, Cape Coast, Ghana; c Placement and Career Services Department, United States International University of Africa, Nairobi, Kenya

**Keywords:** Attitudes, Face mask use, Validation

## Abstract

Despite availability of instruments for measuring attitudes towards mask use, the psychometric properties of many available instruments are not adequately established which limits their research usefulness across contexts. In three studies, we developed the Attitudes Towards Face Mask Use Scale (ATFMUS) in three phases: item generation, scale development, and scale evaluation. Phase one and two were addressed in study 1 while phase three was addressed in studies 2 and 3. In Study 1, a combined online and pen-and-paper sample of 174 (78% university students) completed a questionnaire with 19 items regarding attitudes towards face mask use derived from theory, previous research, and experience. Responses were subjected to item reduction analysis, exploratory factor analysis and reliability analysis. In Study 2, a student sample of 674 (70.5% high school) completed the new scale together with measures of COVID-19 related anxiety and obsession, personality, affect, social media use, and social desirability. Data from the ATFMUS were analyzed using confirmatory factor analysis and pertinent revisions done. The ATFMUS was then validated using correlation analyses, measurement invariance analyses, and known-group comparisons. In study 3, two samples of university students from Ghana (*n* = 242) and Kenya (*n* = 199) were involved in testing the cross-country invariance of the ATFMUS. The results reveal that the 5-item ATFMUS is a reliable and valid scale for assessing attitudes towards face mask use. Invariance analysis revealed that the ATFMUS is fair to use across participants of different age, level of education, and countries. The scale is also sensitive to participants’ actual use of face masks as well as their beliefs about COVID-19 and efficacy of the facemasks. This study offers a foundation for further psychometric evaluation of the ATFMUS.

## 1 Introduction

The global crisis of the Coronavirus disease (COVID-19) led to widespread adoption of several personal protective behaviours as part of the comprehensive prevention and control measures recommended by the World Health Organization (WHO) to contain the spread of the severe acute respiratory syndrome coronavirus 2 (SARS-CoV-2), the virus that causes COVID-19. Many nations made it mandatory to wear face masks in addition to enforcing a range of other non-pharmaceutical preventive measures like: international and domestic travel restrictions, enforcing lockdowns and curfews, closing educational institutions and non-essential businesses, encouraging people to work from home, and limiting public gatherings (WHO, 2020). By mid-2021, the number of COVID-19 cases dropped and there was widespread COVID-19 vaccination roll outs. These dynamics led to relaxation of most COVID-19 prevention measures across the globe (Menni et al., 2022).

With increasing evidence of vaccine effectiveness in preventing COVID-19 infections (Krueger et al., 2022; Menni et al., 2022; Mohamed et al., 2022; Zheng et al., 2021) people's freedom was restored and economies reopened. However, this almost coincided with a resurgence of COVID-19 cases in different countries owing to the emergence of the delta [B.1.617.2] and omicron [B.1.1.529] variants. Additional evidence on vaccine compromisability by the SARS-CoV-2 variants and waning immunity prompted the administration of booster doses to restore vaccine effectiveness (Menni et al., 2022). However, there are a number of vaccination challenges that may contribute to the prolonged pandemic such as disproportionate vaccination coverage, vaccine hesitancy, and vaccine refusal (Wells and Galvani, 2022). This has led to fresh questions on whether communities should be encouraged or required to use face masks and for how long (Bartsch et al., 2022). The value of continuing face mask use lies in their cost-effectiveness and cost saving in efforts to prevent future transmission of SARS-CoV-2 and its variants especially in indoor settings characterized by widespread transmission, difficulties in social distancing, and poor ventilation (Bartsch et al., 2022; WHO, 2021).

Science shows that public face mask use (PFMU) controls community spread of COVID-19 in two ways: First, through source control which involves blocking an infected person's exhalation of infected droplets into the air. Second, it protects uninfected people by creating a barrier that filters out respiratory droplets, particles, and aerosols from inhaled air (Howard et al., 2021; Meyerowitz et al., 2021). Despite initial doubts about the effectiveness of community PFMU (Bundgaard et al., 2021; Greenhalgh et al., 2020; Nanda et al., 2021), there is compelling evidence that mandatory PFMU reduced COVID-19 cases, infection rates, and deaths (Brooks and Butler, 2021) with simulation evidence pointing at the importance of mask use in containing the spread of COVID-19 post vaccination (Bartsch et al., 2022; Kinyili et al., 2022).

Recommendations for continued face mask use are aligned with the precautionary principle that its benefits far outweigh its risks (Greenhalgh et al., 2020; Nanda et al., 2021). In addition, any reduction in community transmission cushions healthcare systems from extra COVID-19 related demands like hospital beds, intensive care unit (ICU) facilities, and ventilators (Greenhalgh et al., 2020; Sousa et al., 2020). Despite the widespread awareness of the role of PFMU to prevent COVID-19, many people resisted wearing face masks or used them wrongly, and others abandoned face mask use all together after vaccination (Bartsch et al., 2022; Esmaeilzadeh, 2022; Mallinas et al., 2021; Pal and Yadav, 2022; Taylor and Asmundson, 2021; Sikakulya et al., 2021). This resistance may have indicated three things: First, that awareness, instructions, threats, and motivation did not necessarily translate into the desirable health behavior of consistent and proper PFMU (Kelly and Barker, 2016; Michie and West, 2021; Tadesse et al., 2020); Second, that people developed psychological reactance to mandatory PFMU (Rosenberg and Siegel, 2018; Taylor and Asmundson, 2021); and third some people had anti-mask attitudes (Mallinas et al., 2021) which lowered the likelihood of PFMU.

With the continuing uncertainty surrounding the pandemic, the value of face masks has been emphasized for all countries across the world (Bartsch, 2022). Consequently, calls have emerged to encourage people to use face masks to prevent future spread of Covid-19 depending on its character just like they use “umbrellas against the rain” or “a coat against the cold” (Martinelli et al., 2021, p. 3). Persuaded by the fact that attitudes and beliefs influence COVID-19 prevention behaviours including intentions (Esmaeilzadeh, 2022; Rieger, 2020; Sun et al., 2021) and that governments currently desire their people to adopt pro-mask attitudes even when mask use is not mandatory (Esmaeilzadeh, 2022; Mallinas et al., 2021), we deem it necessary to have psychometrically sound instruments for assessing attitudes towards PFMU so as to inform mask-use-interventions aimed at reducing COVID-19 transmission. To this end, this study proposes a scale to assess attitude towards face mask use (ATFMUS) and initially validates it among college and high school students in Kenya and Ghana.

### 1.1 Defining attitudes towards face mask use

Aligned with the view by Ajzen (1991), we defined attitudes as a person's positive or negative assessment of public face mask use. We also adapted Mallinas et al. (2021) view that mask use attitudes have two sides: pro-mask use and anti-mask use attitudes. Pro-mask use attitudes involve having positive regard for people who wear masks and negative views of those not wearing masks. On the other hand, anti-mask use attitudes involve having negative views of people who wear masks, and positive views of those not wearing masks. According to Mallinas et al. (2021) distinguishing between pro-mask use and anti-mask use attitudes enables creation of different items for factors that determine support for versus opposition to mask use.

### 1.2 Assessing attitudes towards face mask use

Based on previous works on barriers and concerns in mask-wearing, Esmaeilzadeh (2022) proposes an integrated model with eight sources of attitudes toward mask wearing during the Covid-19 pandemic. The eight concerns are put under three categories that seem to interact in ways that shape general attitudes toward mask-wearing. The three categories are discomfort (physical and communication), external factors (overstated news about coronavirus threat, political beliefs, and absence of mask-wearing culture) and usability issues (lack of effectiveness, unnecessariness of masks in certain cases, and mask maintenance issues). The model further proposes that attitude toward mask-wearing is an important predictor of continuance intention to wear masks. Literature shows that the factors under the three categories are crucial in shaping face mask attitudes and use (Wismans et al., 2022).

Based on the theory of planned behavior (Ajzen, 1991, 2020) people who hold favourable attitudes and subjective norms regarding wearing masks are more likely to form a favourable intention and those who intend to use masks are more likely to do so than those who do not intend to. Although intentions do not always translate into behavior (Ajzen, 2020), measuring attitudes towards mask use can show the public's reactions to mask adoption during the Covid-19 (He et al., 2021) including psychological reactance (Rosenberg and Siegel, 2018; Taylor and Asmundson, 2021). In addition, attitudes are based on the beliefs that people hold regarding the consequences of mask use, others' expectations, and the factors that may facilitate or hinder mask use (Ajzen, 2020; Martinelli et al., 2021). A proper understanding of the attitudes towards mask use has been termed critical in informing national and international PFMU policies (Martinelli et al., 2021) and in enhancing future pandemic preparedness and planning.

Despite remarkable evidence of the role of PFMU in controlling community spread of Covid-19 (Howard et al., 2021; Rieger, 2020), only few scales exist for measuring attitudes towards face mask use. Here are some of the scales we came across:

1. Taylor and Asmundson (2021) developed a 12-item scale assessing negative attitudes about facemasks. The authors constructed the scale to establish how attitudes relate to masks non-adherence and a person's political conservatism. They also evaluated how anti-mask attitudes related to each other. The scale includes five clusters of items: (A) Beliefs that masks are ineffective and possibly harmful, (B) beliefs that mask wearing is an inconvenient habit to form, (C) beliefs that masks are esthetically unappealing, (D) beliefs that masks have adverse interpersonal effects, and (E) beliefs about the physical inconvenience of masks (i.e., difficulty breathing and overheating). Network analyses (Glasso networks using the R qgraph) in a sample of 2,078 adults (age >18 years) from the United States (*N* = 1,036) and Canada (*N* = 1,042) indicated that this scale was reliable.
2. Tadesse et al. (2020) constructed a 9-item attitude towards face mask utilization scale. This was used among police health professionals in Addis Ababa, Ethiopia. However, the authors did not perform any scale evaluation procedures making it rather difficult to adapt.
3. Rieger (2020) used five items to illustrate attitudes and expectations regarding use of facemasks. The items focused on personal aversion to wearing face masks, whether a person would be afraid of others' judgment when wearing a face mask, how well the person thinks wearing a mask protects them from contracting the virus, the perceived effectiveness of wearing face masks in protecting others and the likelihood of wearing masks in public and in other situations. Although this scale has a great potential in measuring attitudes towards face mask use, its psychometric validation was not reported.
4. Mahalik et al. (2022) developed seven questions to assess participants' attitudes toward wearing face-masks or facial coverings. Example items include “I am comfortable being seen wearing a mask,” “Wearing a mask shows that you are scared,” and “I feel wearing a mask is part of my civic duty to protect others.” Upon reverse coding three items, interpretation was in terms of the total score with higher scores indicating more positive attitudes toward mask-wearing. Participants were asked to indicate their level of agreement on a scale of 1 (*Strongly Disagree*) to 6 (*Strongly Agree*) for each statement. The scale had a good level of internal consistency (α = .77) but no other information is available regarding its psychometric validation making it difficult to adapt.

Although the above scales served their purposes well in their respective studies, majority of them present limited information regarding how they were developed. In addition, the existing validated scales on attitudes towards mask use have used different items and analytic approaches. Furthermore, the dimensions of the existing scales are different with some showing a single dimension and others multiple dimensions. Despite the availability of studies on attitudes towards mask use, the psychometric properties of the used instruments are apparently not adequately established which may limit their usefulness in future research on attitudes towards PFMU across contexts. We set out to develop the attitudes towards face mask use scale (ATFMUS) and validated it using college and high school samples drawn from Kenya and Ghana. We developed the AFTMUS in three phases: item generation, scale development, and scale evaluation. Phase one and two were addressed in study 1 while phase three was addressed in studies 2 and 3.

#### 1.2.1 Study 1: ATFMUS item development

Using a rational-theoretical approach (Hubley and Zumbo, 2013) we constructed ATFMUS by reviewing relevant literature (e.g. Rieger 2020; Sikakulya et al., 2021; Taylor and Asmundson, 2021) and by consulting experts on attitudes towards health-related personal protective equipment. A pool of 19 items was identified based on the psychology of attitudes towards use of health protective personal equipment under the following three categories: (1) perceptions on usefulness and effectiveness of face masks (e.g. lack of effectiveness, unnecessariness of masks in certain cases), (2) behaviours related to facemask use in the context of COVID-19 prevention (e.g use of masks, recommending a mask to a friend; absence of mask wearing culture), (3) opinions about physical and interactional side effects of facemasks (e.g. interpreting face mask use as attracting attention, appealing, attractive, different, (un)comfortable). The three categories are well aligned to the model proposed by Esmaeilzadeh (2022).

### 1.3 Methods

#### 1.3.1 Participants and procedure

We involved a combined online and pen-and-paper sample of 174 participants (51% female) with an average age of 25.99 years (*SD* = 7.10; range = 14 to 60). The online sub-group comprised of 103 participants (53% women) with an average age of 26.13 years (*SD* = 5.99; range = 17–49 years). Majority were university students (78%) and the rest were either in college (17%) or high school (5%). Data were collected during the months of May and June 2020. Owing to the COVID-19 lock down in Kenya in the period of data collection, the link for the online questionnaire was mainly distributed via student Whatsapp groups. This sub-group had participants from 23 out of 47 counties in Kenya. We expected a larger online sample but we obtained a low return rate. A follow up through the groups established that the cost of data bundles against the backdrop of the harsh economic changes due to COVID-19 put off many respondents from filling the online questionnaire. Consequently, a pen-and-paper sample was added to enhance representation in the study. This sub-group was mainly drawn from a driving school in Murang'a County comprising of 71 participants (54% male) with a mean age of 25.80 (*SD* = 8.49; range = 14 to 60). Almost half of this group (48%) had university level of education with the rest having college (27%) and secondary school (24%) levels of education. Participation in the study was voluntary and all participants gave informed consent before completing the questionnaire.

#### 1.3.2 Instruments

Participants filled a questionnaire whose first part included demographic questions. Beliefs about COVID-19 and face mask use were assessed through three face valid yes/no items: “Do you believe there is COVID-19?”; “Do you always wear a face mask in public?”); and belief about the efficacy of face masks (“can a face mask prevent COVID-19?”). Participants then completed a 19-item scale designed for this study to assess attitudes towards face mask use. Each item was rated on a 5-point Likert scale ranging from 1 = *Strongly Disagree* to 5 = *Strongly Agree*. The questionnaire comprised of items that collected demographic data, and attitudes towards face mask use.

### 1.4 ATFMUS item evaluation

Based on the classical test theory (CTT), each item was first evaluated individually, then exploratory factor analysis was done, and finally internal consistency reliability for the scale was established. We provide more details of these analyses in the following section.

#### 1.4.1 Step 1 Preliminary item reduction analysis

In this step, we evaluated the descriptive statistics for each of the 19 items: mean, the standard deviation, skewness, kurtosis, and inter-item correlations (see Table 1). According to Lester et al. (2014) item means and variances provide preliminary evidence of whether an item can provide useful information or not. In Likert scales where scores range from 1 to 5, a mean of 3.0 and a standard deviation of 1.0 are the most ideal since they ensure a reasonable distribution of responses along the scale. An item with a very high mean of above 4.5 or a very low mean (1.5 and below) and low variability (standard deviation less than 1.0) would suggest a skewed item (either left or right) and such an item is less useful. As observed by Jin et al. (2018), there are no specific criteria for item level analysis using these descriptive statistics. We used the most tolerant exclusion criterion for item means adopted by Jin et al. (2018) defined by lowest score option plus 20% of the score range and the highest score option minus 20% of the score range. Since we used a 5-point Likert scale, each item in the scale had a range of 4 implying that the exclusion criterion based on item means was lower than 1.8 or higher than 4.2. With regards to item score *SD*, an item would be excluded if its *SD* was smaller than one-sixth of the score range (i.e. 1/6 × 4 = 0.67).

**Table 1:** Descriptive statistics for the ATFMUS items.

Item	M	SD	Skewness	Kurtosis	CITC
1. Face masks are uncomfortable^10^	3.74	1.46	−0.87	−0.69	.40
*2. The idea of using face masks is not appealing*^*5,10*^	2.83	1.51	0.09	−1.45	.55
3. Proper use of face masks can enhance one's safety from the coronavirus	4.34	1.15	−1.75	1.99	.00
4. I intend to use always use face masks as long as there is the coronavirus	4.28	1.19	−1.70	1.18	−.02
5. I would be comfortable suggesting to a friend to use a face mask	4.21	1.25	−1.53	1.11	−.15
*6. I would avoid using a face mask if possible*^*5,10*^	2.75	1.67	0.19	−1.66	.44
*7. I just don't like the idea of using face masks*^*5,10*^	2.70	1.52	0.23	−1.45	.49
8. People who use face masks show concern and responsibility to those around them	4.53	0.98	−2.35	4.95	.12
9. Using face masks is unfashionable	2.19	1.50	0.88	−0.75	.24
10. A face mask is the best way to protect myself from the coronavirus	3.56	1.38	−0.58	−0.94	−.20
11. It is polite to wear a face mask in public	4.52	0.94	−2.32	5.20	−.10
12. Wearing a face mask may make friends think that you have the coronavirus	1.53	1.01	2.07	3.67	.18
*13. I only wear a face mask when I know I am likely to be punished for not wearing one*^*5,10*^	2.09	1.48	0.90	−0.86	.36
*14. Face masks are unhygienic*^*5,10*^	1.99	1.29	0.91	−0.56	.35
15. People are likely to stare at me more when I wear a face mask^10^	1.96	1.31	1.05	−0.38	.34
16. Sometimes I don't wear a face mask so that I can be like the people near me^10^	1.99	1.47	1.09	−0.47	.29
17. A face mask makes breathing difficult^10^	3.61	1.39	−0.81	−0.66	.43
18. Wearing face masks may not change anything because there is no coronavirus	1.40	0.93	2.49	5.43	.26
19. As long as I socially distance, I don't need a face mask∗^10^	1.97	1.44	1.11	-0.38	.27

*Note. N* = 174. *M =* Mean*; SD =* Standard Deviation*, CITC =* Corrected Item-total Correlation.

^5^Italicized items retained in the final 5-item ATFMU scale; ^10^Item retained in the 10-item ATFMU scale.

∗Item had CTIC <.30 but was retained in the scale owing to its importance in exploring unnecessariness of masks.

These criteria were consistent with those reported in scale development literature (Jin et al., 2018; Lester et al., 2014). In CTT, item discrimination and difficulty indices are typically used to decide which items should be retained and which ones should be discarded or replaced (Streiner et al., 2015). Item difficulty is typically indexed by the mean score on the item. The most common index of item discrimination is the correlation between the item score and the item-total correlation (Boateng et al., 2018; Haladyna, 2016; Lester et al., 2014). Using data from the 174 participants, we computed measures of distribution for each item which enabled us to perform item reduction analysis. We followed two criteria for item removal: First, all items with kurtosis and skewness higher than |2| were to be removed as they were deemed not to be normally distributed (Kline, 2015; Nguyen, 2019). Second, we computed the corrected item-total correlations and items with correlations below .3 (“very low correlations”; Boateng et al., 2018) were candidates for removal. In ATFMUS, four items were removed due to kurtosis and skewness values higher than |2| and five items were removed due to item-total correlations below .30. Although item 19 had an inter-item correlation of .27 it was retained due to its relevance in measuring unnecessariness of masks. Incidentally, all the removed items had means above 4.2. This ensured that only those items that were parsimonious, functional, and internally consistent were ultimately included (Boateng et al., 2018).

To further understand the items that make up the ATFMUS, we performed a cross-correlation of all the items as separate variables. According to scale development literature, this step is necessary as it helps in illustrating clusters of interrelating items Boateng et al (2018). When all items in a scale post significant inter-item correlations, it reveals that they assess aspects of the same attribute (Coaley, 2010). The inter-item correlations are also initial indicators of the structural validity of a scale (Yoo and Pituc, 2013). Those items that failed to inter-correlate significantly were candidates for removal. The inter-item correlations for the ATFMUS were as presented in Table 2.

**Table 2:** Inter-item correlations for the ATFMUS.

Item	1^10^	2^5,10^	3	4	5	6^5,10^	7^5,10^	8	9	10	11	12	13^5,10^	14^5,10^	15^10^	16^10^	17^10^	18	19^10^
2	.44∗∗	-																	
3	−.01	.09∗	-																
4	.01	.11∗∗	.46∗∗	-															
5	.07	.15∗∗	.44∗∗	.52∗∗	-														
6	.32∗∗	.36∗∗	.14∗∗	.14∗∗	.15∗∗	-													
7	.25∗∗	.40∗∗	.19∗∗	.20∗∗	.23∗∗	.48∗∗	-												
8	−.01	.05	.29∗∗	.23∗∗	.29∗∗	.04	.09∗	-											
9	.14∗∗	.21∗∗	.13∗∗	.16∗∗	.14∗∗	.26∗∗	.22∗∗	.11∗∗	-										
10	.10∗∗	.17∗∗	.38∗∗	.35∗∗	.36∗∗	.18∗∗	.23∗∗	.27∗∗	.13∗∗	-									
11	.02	.07	.32∗∗	.29∗∗	.35∗∗	.07	.12∗∗	.34∗∗	.07	.30∗∗	-								
12	.09∗	.17∗∗	.27∗∗	.28∗∗	.23∗∗	.16∗∗	.23∗∗	.17∗∗	.28∗∗	.23∗∗	.21∗∗	-							
13	.16∗∗	.26∗∗	.24∗∗	.28∗∗	.30∗∗	.33∗∗	.36∗∗	.16∗∗	.20∗∗	.26∗∗	.21∗∗	.32∗∗	-						
14	.14∗∗	.25∗∗	.24∗∗	.19∗∗	.27∗∗	.21∗∗	.31∗∗	.19∗∗	.18∗∗	.26∗∗	.15∗∗	.27∗∗	.27∗∗	-					
15	.10∗∗	.13∗∗	.17∗∗	.17∗∗	.14∗∗	.17∗∗	.14∗∗	.10∗∗	.15∗∗	.18∗∗	.13∗∗	.34∗∗	.22∗∗	.22∗∗	-				
16	.10∗∗	.13∗∗	.08∗	.15∗∗	.11∗∗	.19∗∗	.17∗∗	.16∗∗	.16∗∗	.11∗∗	.06	.22∗∗	.25∗∗	.18∗∗	.31∗∗	-			
17	.22∗∗	.30∗∗	.05	.13∗∗	.09∗	.26∗∗	.27∗∗	-.02	.15∗∗	.12∗∗	-.04	.10∗∗	.31∗∗	.16∗∗	.13∗∗	.16∗∗	-		
18	.09∗	.17∗∗	.28∗∗	.31∗∗	.34∗∗	.16∗∗	.23∗∗	.26∗∗	.19∗∗	.23∗∗	.23∗∗	.37∗∗	.38∗∗	.29∗∗	.23∗∗	.24∗∗	.19∗∗	-	
19	.12∗∗	.27∗∗	.20∗∗	.29∗∗	.26∗∗	.22∗∗	.31∗∗	.15∗∗	.25∗∗	.26∗∗	.20∗∗	.33∗∗	.40∗∗	.27∗∗	.19∗∗	.14∗∗	.25∗∗	.39∗∗	-

*Note*. *N* = 174; ^5^Items retained in the final 5-item ATFMU scale; ^10^Item retained in the 10-item ATFMU scale.

∗*p* ≤ .05. ∗∗*p* ≤ .01.

As presented in Table 2, all items retained in the ATFMUS had significant inter-correlations implying that they were measuring aspects of attitudes towards face mask use (Coaley, 2010).

#### 1.4.2 Step 2 Extraction of factors

To study the underlying structure and estimate the construct validity of the ATFMU scale, we conducted an exploratory factor analysis (EFA, principal components with Varimax rotation). Kaiser-Meyer-Olkin value was 0.82 and Bartlett's test of sphericity was significant (χ^2^_(45)_ = 445.89, *p* < .001), supporting the sampling adequacy and rationale for performing EFA. The initial analyses (see Table 3) yielded a two factor solution with the first factor explaining 26.24 percent of variance and the second factor explaining 24.70 percent of the total variance. We evaluated the statistical meaning of the loadings using the criteria given by Tabachnick and Fidell (2019) of 0.32 (poor), 0.45 (fair), 0.55 (good), 0.63 (very good), and 0.71 (excellent). In the analysis presented in Table 3, all the 10 items had good to excellent loadings ranging from .59 to .78. The results suggested that the obtained solution was a good estimate of a simple structure since all items had strong positive loadings on one factor and small cross loadings on the other factors. The scree plot suggested up to two primary factors, each with eigenvalues above 1. To confirm the possible number of factors that can be extracted from our data, we performed a parallel analysis (Horn, 1965) of 100 random datasets with 174 subjects and 19 variables using the 95% cutoff. The results supported a one-factor solution since the second eigenvalue from the real data (3.66, 1.43) failed to exceed the second eigenvalue in the random data (1.63, 1.51) as per the criteria reported in literature (Çokluk and Koçak, 2016; Lim and Jahng, 2019; O'connor 2000; Patil et al., 2007). We therefore, redid the EFA restricted to a one factor extraction. As presented in Table 3, all the items loaded positively to the resultant factor with loadings ranging from .49 to .72. The one factor explained 36.62 percent of variance.

**Table 3:** Exploratory factor structure and internal consistency reliability.

	1-Factor model			2- Factor model			
Item	Factor 1	CITC	α if item deleted	Factor 1	Factor 2	CTIC	α if item deleted
1	.52	.41	.80	**.78**	−.07	.52	.73
2	.71	.59	.77	**.76**	.23	.63	.69
7	.72	.59	.77	**.70**	.34	.60	.70
6	.65	.52	.78	**.64**	.27	.53	.73
17	.53	.42	.79	**.61**	.12	.41	.76
15	.49	.38	.80	−.04	**.76**	.50	.67
14	.65	.52	.78	.26	**.67**	.51	.67
16	.51	.39	.80	.08	**.66**	.45	.69
13	.64	.52	.78	.28	**.64**	.51	.67
19	.57	.46	.79	.24	**.59**	.45	.69
% variance explained	36.62			26.24	24.70		
*M(SD)*	25.63 (8.77)			15.63 (5.44)	9.99 (4.83)		
α	.80			.77	.73		

*Note. N* = 174; *CITC* = Corrected Item-Total Correlation; *M* = Mean; *SD* = Standard Deviation.

#### 1.4.3 Step 3 Internal consistency reliability

To test the internal consistency reliability we computed the corrected item-total correlation and Cronbach's alpha (Haladyna, 2016). These tests were done at the factor level based on the results of the EFA and parallel analysis in step 2. At this stage, an item was to be removed if its item-total correlation was below .30 and/or if the Cronbach's alpha of the factor increased after removing an item (Hubley and Zumbo, 2013). We tested for the internal consistency reliability for both the one factor model suggested by parallel analysis and the two-factor model suggested by EFA. Both models had good internal consistency reliability with the one-factor model having α = .80, while factors one and two had α = .77 and α = .73 respectively in the two-factor model (see Table 3). All the items met the criteria for retention in the two models.

## 2 Study 2: evaluation of the ATMFUS

### 2.1 Introduction

In Study 2 we set out to validate the ATFMUS and provide further evidence to support its dimensionality and reliability as implied by the EFA and parallel analysis in Study 1. We also included several measures to assess convergent and divergent validity. In this study, we tested several hypotheses: First, we hypothesized that the one factor model may have a better fit than the two-factor model. Secondly, we hypothesized that as a test of convergent validity, the attitudes towards face mask use were related to: a) personality traits; b) affect; and c) COVID related anxiety and obsession (risk perception). Thirdly, as a test for divergent validity, we posited that attitudes towards face mask use were not related to social media use. Fourthly, we hypothesized that there were no differences in the attitudes towards face mask use in terms of gender, level of education, and age group of the participants.

### 2.2 Method

#### 2.2.1 Participants and procedure

The study involved 674 participants who were mainly middle and late adolescents (355 female; mean age = 18.26 years; *SD* = 1.70; range 14–28 years). Eligibility criteria and the procedure matched that used in Study 1. Again, the participants were all Kenyan with majority (70.5%) being in high school. The participants were drawn from Nyeri County (45.5%); Nairobi County (28.9%); Meru County (12%); and Kiambu County (13.6%). Over three quarters of the participants (78.8%) indicated that they believed that there is Covid-19 and 77.4% reported that they wore masks in public. In addition, 61.9% reported that face masks can prevent Covid-19.

The school administrators acted as the legal guardians for all participants below the age of 18 and provided informed consent for their participation. Participants aged 18 and above signed an informed consent form before completing the questionnaires. A member of the research team supervised the participants as they filled the questionnaires in a classroom context. The participants were not given any reward or compensation for their participation.

#### 2.2.2 Measures

**Attitudes towards Face Mask Use Scale (ATFMUS):** The 10-item ATFMUS developed in Study 1 was used to test attitudes towards face mask use. Each item was rated on a 5-point Likert scale ranging from 1 (*Strongly Disagree*) to 5 (*Strongly Agree*).

**Obsession with COVID-19 Scale (OCS):** The OCS (Lee, 2020a) is a self-report mental health screener of persistent and disturbed thinking about COVID-19. This scale consists of four items (e.g., “I had disturbing thoughts that I may have caught the coronavirus”) each rated on a 5-point scale, from 0 (*not at all*) to 4 (*nearly every day*) based on experiences over the past two weeks. Lee (2020a) reported that the OCS is a reliable instrument (with αs > .83), with solid factorial (single-factor) and construct validity (correlated with coronavirus anxiety, spiritual crisis, alcohol/drug coping, extreme hopelessness, and suicidal ideation). The OCS total scores range from 0 to 20 with a total score of ≥7 indicating probable dysfunctional thinking about COVID-19. In the present study, the OCS showed good internal consistency (α = .875).

**Coronavirus Anxiety Scale (CAS):** The CAS (Lee, 2020b) is a self-report tool for assessing dysfunctional anxiety associated with the coronavirus crisis based on experiences over the past two weeks. This scale consists of five items (e.g., “I had trouble falling or staying asleep because I was thinking about the coronavirus”) each rated on a 5-point scale, from 0 (*not at all*) to 4 (*nearly every day*). Lee (2020b) reported that the CAS is a reliable instrument (αs > .90), with solid factorial (single-factor; invariant across sociodemographics) and construct validity (correlated with anxiety, depression, suicidal ideation, and drug/alcohol coping). The CAS total scores range from 0 to 20 with a total score of ≥9 indicating probable dysfunctional coronavirus-related anxiety. In this study, the CAS showed good internal consistency (α = .811).

**Negative and Positive Affect Scale (NAPAS):** We used the 10-item NAPAS (Joshanloo, 2017) to measure affect based on experiences during the past 30 days. This scale comprises of five items that measure negative affect and another five items measuring positive affect. Each item is rated on a 5-point scale, from 1 (*none of the time*) to 5 (*all of the time*). In the present study, the NAPAS showed good internal consistency (α = .811).

**Big Five Inventory- 10 (BFI-10):** The BFI-10 scale (Rammstedt and John, 2007) measures the Big Five personality dimensions. The scale has five subscales each consisting of two bidirectional items with responses ranging from 1 (*strongly disagree*) to 5 (*strongly agree*). After reverse-coding the necessary items, the subscales had unacceptably low reliability coefficients. However, we took consolation from previous studies reporting low subscale alphas (Balgiu, 2018; Carciofo et al., 2016; John et al., 2019) as well as from the disclaimer by Gosling et al. (2022) that it is almost impossible to get high alphas for brief instruments measuring broad domains with only two items per dimension. We tolerated the low reliability of the scale since personality was only used exploratorily in checking the validity of the ATFMU scale.

**The Bergen Social Media Addiction Scale (BSMAS):** This scale is an adaptation of the Bergen Facebook Addiction Scale (BFAS; Andreassen et al., 2012). It consists of six items reflecting major addiction components. Each item is concerned with experiences during the past year (e.g., “How often during the last year have you used social media to forget about personal problems?”) and it is rated on a 5-point Likert scale ranging from *very rarely* (1) to *very often* (5) resulting in composite scores ranging from 6 to 30. In this study, BSMAS had a good internal consistency (α = .62).

**Social Desirability–Gamma Short Scale (KSE-G):** The English-language KSE-G (Nießen et al., 2019) is an adaptation of the Kurzskala Soziale Erwünschtheit–Gamma (KSE-G, Kemper et al., 2014) measuring two aspects of the Gamma factor of socially desirable responding (SDR): exaggerating positive qualities (PQ+) and minimizing negative qualities (NQ−). It consists of six items rated on a 5-point scale ranging from 1 (*doesn't apply at all*) to 5 (*applies completely*). Each subscale's total score ranges from 3 to 15. The unweighted mean score of the three items of each subscale is computed. Nießen et al. (2019) reported reliability estimates ranging from α = .64 to α = .79 across three samples. The scale also showed sufficient construct validity by revealing an underlying moralistic bias in answering personality items (through correlations with the Big Five Personality traits). In this pilot study, the KSE-G showed low internal consistency estimates (PQ+, α = .578; NQ-, α = .471) but we decided to retain the scale to explore moralistic bias in answering the ATFMUS items.

### 2.3 Data analysis

#### 2.3.1 Factor structure

We used confirmatory factor analysis (CFA) to evaluate the factor structure of the ATFMUS. First, the data were fitted to the two-factor and the one-factor models suggested by EFA and parallel analysis (Horn, 1965) respectively in Study 1. The criteria for assessing model fit in the CFA was based on five indices: A Chi square measure of fit, Chi-square statistic (χ^2^) and its *p*-value >.05, χ^2^/*df* of 3 or less, Root Mean Square Error of Approximation (RMSEA ≤0.06), Comparative Fit Index (CFI ≥0.95) and Standardized Root Mean Square Residual (SRMR) of 0.09 or less (Hu and Bentler, 1999; Iacobucci, 2010).

#### 2.3.2 Reliability

The reliability of the ATFMUS was established using Cronbach's alpha. According to the good practice guideline by Boateng et al. (2018), an alpha of .70 or higher is acceptable.

#### 2.3.3 Measurement invariance

We further tested for measurement invariance for the scale using AMOS Version 26 to ensure that the same construct was being measured across groups. We tested two levels of variance in two steps: 1. Configural invariance where thresholds and factor loadings were free across groups. Residual variances were fixed at one in all groups, and factor means were fixed at zero in all groups. It was the least constrained model. 2. Metric invariance where thresholds and factor loadings were constrained to be equal across groups. As a default, residual variances were fixed at one in the first group and freely estimated in the second group. Factor means were fixed at zero in the first group and freely estimated in the second group. It was the more constrained model (Kline, 2015; Little, 2013). A chi-square difference tester the “χ^2^ Difference” (Gaskin, 2016) evaluated the difference between the unconstrained and constrained models. Measurement invariance was inferred if the chi-square difference value was not statistically significant. We tested for invariance in four steps (Kline, 2015; Little, 2013; Widaman and Grimm, 2014): Configural level where all parameters are freely estimated, and each indicator's factor loadings for all groups are checked for significance, metric level where each indicator's factor loading is constrained to be equal across groups, and the model fit is compared with that of the configural invariance model, and scalar level where each indicator's intercept is constrained to be equal across groups, and the model fit was compared with that of the metric invariance model. Measurement invariance was inferred from a ΔCFI ≤.01 and a ΔRMSEA ≤.015 (Chen, 2007; Cheung and Rensvold, 2002; Kline, 2015).

#### 2.3.4 Construct validity

To evaluate the construct validity of the ATFMUS, we used the four indicators suggested by Boateng et al. (2018): convergent validity, discriminant validity, differentiation by known groups (gender, level of education, age) and correlation analysis.

### 2.4 Results

#### 2.4.1 ATFMUS factor structure

**Figure 1:**
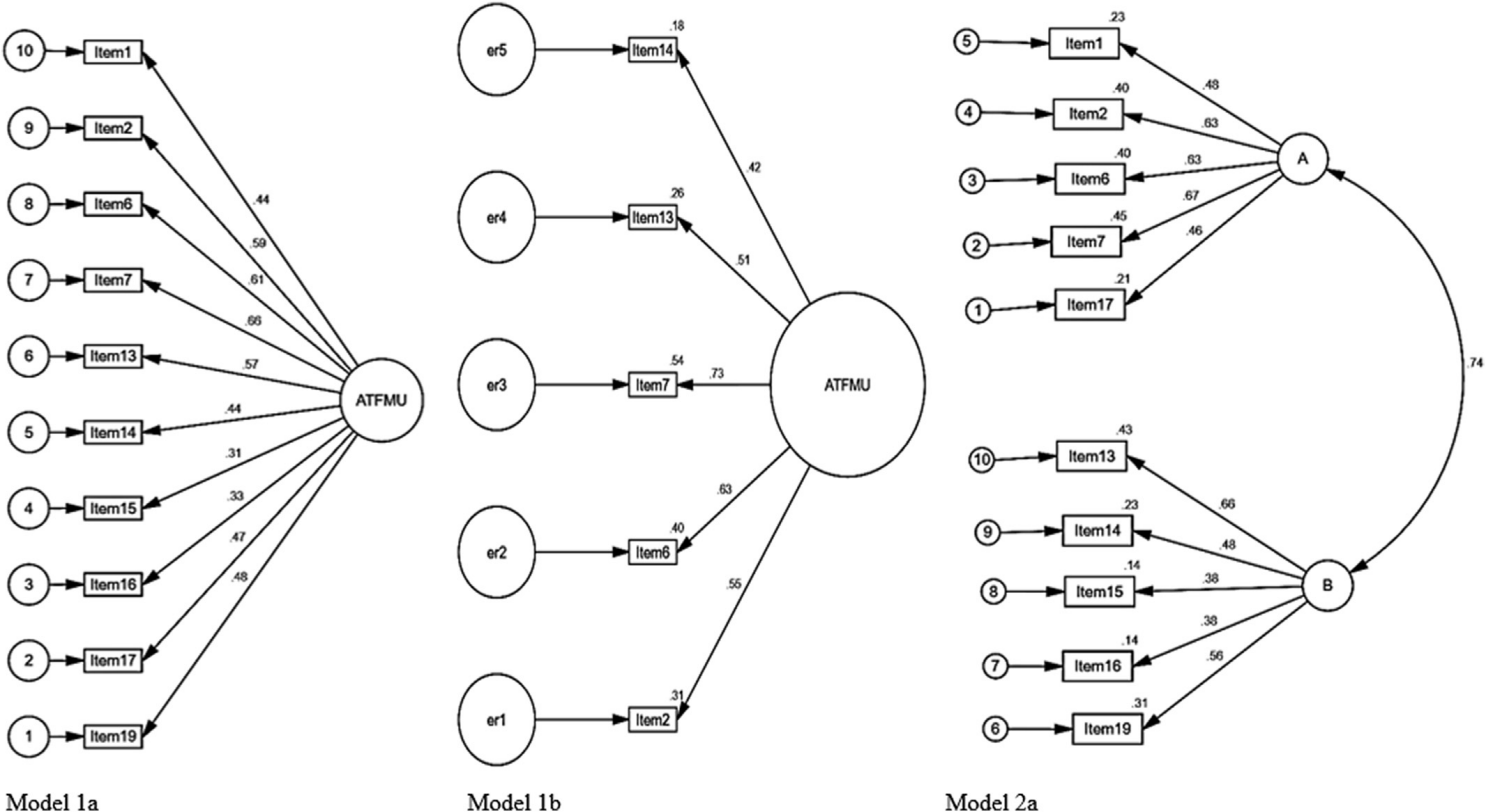
ATFMUS factorial structure.

We tested the factorial structure of three models using CFA (see Figure 1 and Table 4). Model 1a was a one-factor model (comprising of 10 items) and it turned out as having lesser satisfactory fit to the data: χ^2^ (35) = 192.834; *p* = .000; χ^2^/*df* = 5.51; GFI = .94; CFI = .86; RMR = .14; TLI = .82; RMSEA = .08, 90 CI [.07,.09]. Model 2a was a correlated two-factor model (comprising of 10 items). This model had a better fit than Model 1a: χ^2^ (34) = 129.176; *p* = .000; χ^2^/*df* = 3.80; GFI = .96; CFI = .92; RMR = .11; TLI = .89; RMSEA = .06, 90 CI [.05,.08]. However, the model did not quite meet the desired values for acceptable fit. Through fit checks on standardized residual covariances five items were iteratively dropped yielding a best fit model (1b) with χ^2^_(5)_ = 9.89; *p* = .08; χ^2^/*df* = 1.98; GFI = .99; CFI = .99; RMR = .056; TLI = .98; RMSEA = .04, 90 CI [.00,.08]. Given these results, the ATFMUS was better conceptualized as a 5-item one factor scale rather than a two-factor scale. The items retained in ATFMUS all focused on negative aspects of facemask use and therefore the scale may function well in capturing negative attitudes towards face mask use.

**Table 4:** Model fit indices for ATFMUS.

Model	Items	χ^2^	DF	*p*	χ^2^/DF	RMSEA [90% CI]	RMR	GFI	CFI	TLI
1a	10	192.834	35	.00	5.51	.08 [.07, .09]	0.14	.94	.86	.82
2a	10	129.176	34	.00	3.80	.06 [.05, .08]	0.11	.96	.92	.89
1b	5	9.888	5	.08		.04 [.00,.07]	0.06	.99	.99	.98

#### 2.4.2 Reliability

We then tested the reliability of the final version of the ATFMUS using Cronbach's alpha with the 5-item scale yielding α = .71. This was sufficient as per the good practice guideline by Boateng et al. (2018) who argue that an alpha of .70 or higher is acceptable.

#### 2.4.3 Measurement invariance

We further explored how the dimensionality of the ATFMUS generalized across participants grouped in terms of gender, level of education, and age. The measurement invariance results were as given in Table 5.

**Table 5:** Measurement invariance of ATFMU across sex, level of education and age group.

Model	χ^2^ (df)	CFI	RMSEA [90% CI]	SRMR	Model	ΔCFI	Δ χ^2^ (Δdf)	ΔRMSEA	Δ SRMR	Invariant?
*Sex (*Male, *n* = 319; Female, *n* = 355)										
Configural	28.151∗∗ (10)	.967	.052 [.030, .075]	.014		--	--	--	--	
Metric	32.901∗∗(14)	.966	.045 [.025, .065]	.026		−.001	4.750 (4)	−.007	.012	Yes
Scalar	34.558∗ (18)	.970	.037 [.017, .055]	.026		.004	1.657 (4)	−.008	.000	Yes
Residual	39.932∗(23)	.969	.033 [.014, .050]	.032		−.001	5.558 (5)	−.004	.006	Yes
*Age group* (≤18 years, *n* = 381, ≥19 years, *n* = 293)										
Configural	10.753 (10)	.999	.011 [.000, .044]	.024		--	--	--	--	
Metric	16.616 (14)	.995	.017 [.000, .043]	.030		−.004	5.863 (4)	.006	.006	Yes
Scalar	18.941 (19)	1.000	.000 [.000, 0.34]	.031		.005	2.325 (5)	−.017	.001	Yes
Residual	24.636 (24)	.999	.006 [.000, .032]	.036		−.001	5.695 (5)	.006	.005	Yes
*Level of Education* (Secondary, *n* = 475, University, *n* = 199)										
Configural	19.103∗(10)	.983	.037 [.008, .062]	.023		--	--	--	--	
Metric	21.621 (14)	.986	.028 [.000, .051]	.024		.003	2.518 (4)	−.009	.001	Yes
Scalar	23.840 (19)	.991	.019 [.000, .041]	.024		.005	2.219 (5)	−.009	.000	Yes
Residual	28.076 (24)	.992	.016 [.000, .037]	.026		.001	4.236 (5)	−.003	.002	Yes

*Note. N* = 674.

∗ *p* ≤ .05. ∗∗ *p* ≤ .01

##### 2.4.3.1 Gender

In testing measurement invariance, we started by establishing the baseline (configural) model (based on Model 1b) which had good fit to the data: TLI = .935, CFI = .967, RMSEA = .052, SRMR = .014. In the second step, we compared the metric model (with loadings constrained to be equal) to the configural model. The change in χ^2^ was not significant, Δχ^2^(4) = 4.75, *p* = .314, which indicated that the loadings between the two groups were similar. In the third step, we tested for scalar invariance and the change in χ^2^ was also not significant Δχ^2^(8) = 6.41, *p* = .602. In addition, this finding showed that the thresholds were similar across gender. In addition, the CFI decreased by .001 (Metric vs. Configural), and then increased by .004 (Scalar vs. Metric), and the RMSEA decreased by .007 and .008 respectively for both comparisons. These results showed convincingly ATMFUS is fair when comparing male and female participants.

##### 2.4.3.2 Age

The configural model for age (middle adolescents vs late adolescents) had good fit to the data: TLI = .997, CFI = .999, RMSEA = .011, SRMR = .024. Metric invariance was supported by the fact that constraining the loadings to be equal did not lead to a significant change in Chi square (Δχ^2^ (4) = 5.863, *p* = .210) which meant that the two groups had similar loadings. In addition, scalar invariance was indicated by the fact that constraining the intercepts to be equal across the groups did not lead to a significant change in χ^2^, Δχ2 (9) = 8.188, *p* = .515. Here, the CFI decreased by .004 (Metric vs. Configural), and then increased by .005 (Scalar vs. Metric), and the RMSEA increased by .006 and then decreased by .017 respectively for both comparisons. These results showed convincingly ATMFU scale has the same meaning to the middle and late adolescents.

##### 2.4.3.3 Level of education

The configural model for testing for invariance in terms of level of education (secondary vs university) had a good fit to the data: CFI = .983, RMSEA = .037, SRMR = .023. Upon constraining the loadings to be equal across the groups, the change in χ^2^ was not significant Δχ^2^ (4) = 2.518, *p* = .641 supporting metric invariance. Scalar invariance across the groups was supported by the fact that constraining thresholds did not lead to a significant change to χ^2^, Δχ^2^ (9) = 4.737, *p* = .857.

##### 2.4.3.4 Known-group validity

To examine the sensitivity of the ATFMUS, we conducted twelve known group comparisons using independent samples t-test. We first compared the ATFMUS scores between participants by gender, then level of education, and by age grouped as ≤18 and ≥19 years. Cohen's *d* effect sizes were calculated as either trivial (<0.2), small (≥0.2 and <0.5), moderate (≥0.5 and <0.8) or large (≥0.8) (Cohen, 1988). We then compared the ATFMUS scores across participants in terms of whether they answered “yes” or “no” to the following three questions: “Do you always wear a face mask when you are in public?”, “Do you believe there is coronavirus?”, and “Can a face mask prevent coronavirus?”. Table 6 summarizes the findings.

**Table 6:** Group comparisons of the ATFMUS.

	Group	n	M (SD)	t	df	Sig.	Cohen's d
Gender	Male	319	15.52 (5.22)	.54	672	.59	.04
	Female	355	15.30 (5.45)				
Level of education	Secondary	475	15.34 (5.33)	−.51	672	.61	.04
	University	199	15.57 (5.36)				
Age	≤18 years	381	15.20 (5.46)	−1.12	672	.26	.09
	≥19 years	293	15.67 (5.17)				
Always wear a face mask in public?	Yes	522	16.06 (5.28)	6.06	672	.00	.54
	No	152	13.15 (4.90)				
Believe there is Corona Virus?	Yes	541	16.16 (5.16)	7.40	672	.00	.67
	No	143	12.58 (5.03)				
Face mask can prevent Corona Virus?	Yes	417	16.78 (5.12)	8.94	671	.00	.67
	No	256	15.67 (4.91)				

We first compared the ATFMUS total scores of male and female participants using an independent samples t-test. No statistically significant gender difference was found. The Cohen's *d* effect size was 0.04. Again no statistically significant differences were found when we compared the ATFMUS total scores by level of education (*d* = 0.04), and by age group (*d* = 0.09). Across these groups the effect sizes were all trivial. On the other hand, we found statistically significant differences in terms of whether or not the participants always wore masks in public (*d* = .54), believed there is Corona Virus (*d* = .67) and whether they agreed that face masks can prevent COVID-19 (*d* = .67). The effect sizes across the last six groups were moderate.

##### 2.4.3.5 ATFMUS correlations with other well established scales

To establish convergent and discriminant construct validity, we evaluated the correlations between the ATFMUS composite score and other well established scales (see Table 7). The results revealed significant positive correlations between ATFMUS and consciousness (*r* = .22) and agreeableness (*r* = .17); exaggerating of positive qualities (*r* = .13); obsession with Covid-19 (*r* = .10), and Corona Anxiety (*r* = .08). On the other hand, ATFMUS had significant negative correlations with minimizing negative qualities (*r* = −.15); and negative affect (*r* = −.11).

**Table 7:** Pearson correlations between ATFMUS and other well established scales.

Scale	ATFMUS
Obsession with COVID-19 Scale (OCS)	.10∗∗
Coronavirus Anxiety Scale (CAS)	.08∗
Negative Affect- PANAS	−.11∗∗
Positive Affect- PANAS	.03
The Bergen Social Media Addiction Scale (BSMAS)	−.05
Extroversion (BFI-10)	−.04
Agreeableness (BFI-10)	.05
Conscientiousness (BFI-10)	.13∗∗
Neuroticism (BFI-10)	.04
Openness (BFI-10)	.02
Social Desirability: Exaggerating positive qualities (KSE-G PQ+)	.06
Social Desirability: Minimizing negative qualities (KSE-G PQ-)	−.15∗∗

*Note.* ATFMUS = attitudes towards face mask use scale; KSE-G = Social Desirability–Gamma Short Scale.

∗*p* < 0.05. ∗∗*p* < 0.01*.*

## 3 Study 3: cross-country invariance of ATFMUS

### 3.1 Introduction

There is evidence of cross-country differences in citizens’ attitudes towards government pandemic policies (Brouard et al., 2022) and towards Covid-19 interventions like vaccines (Greyling and Rossouw, 2022). Such differences may indicate that the factors influencing how people develop, express, and maintain attitudes vary with environment, culture, and social experience. Considering the critical role of attitudes towards COVID-19 interventions in efforts aimed at winning the global fight against COVID-19, there is need to keep refining efforts aimed at effectively assessing such attitudes If the ATFMUS is to be put to meaningful cross-cultural use, then there must be evidence that it measures the same variable the same way across the cultural contexts (Boateng et al., 2018; Streiner et al., 2015). In this study, we sought to test whether the hypothesized unidimensional structure of the ATFMUS is the same across student samples obtained from Ghana and Kenya (configural invariance). We also investigated whether test items measure the attitudes towards face mask use equally across the countries (metric invariance); and whether individuals from different countries who get similar ATFMUS scores actually possess similar attitudes towards face mask use (scalar invariance).

### 3.2 Method

#### 3.2.1 Participants and procedure by country

**Ghanaian sample:** The Ghanaian sample comprised of 242 undergraduate students (59.5% female) from the University of Cape Coast who filled the 5-item ATFMUS (confirmed in study 2) as part of a larger study. Potential participants were recruited from students attending face-to-face lectures in the university in the academic year 2020/2021. The total sample had an average age of 22.73 (*SD* = 4.28; range = 17–45 years). The average age for the male participants was 23.78 (*SD* = 5.17) while that of the female participants was 22.01 (*SD* = 3.39). The male participants were significantly older than female participants *t* (240) = 3.20, *p* = .002. The study received approval from the ethics board from the Ghanaian author's research institution, and all participants gave an informed consent for participation in the study.

**Kenyan sample:** The Kenyan sample comprised of 199 undergraduate students (57.8%) from Kenyatta University who filled the 5-item ATFMUS as part of a larger study on links between personality and academic outcomes. Participants were recruited from those attending face-to-face lectures during the second semester of 2020/2021. The total sample had an average age of 19.60 (*SD* = 1.41; range = 17–28 years). The average age for the male participants was 20.11 (*SD* = 1.59) while that of the female participants was 19.23 (*SD* = 1.14). The male participants were significantly older than female participants *t* (197) = 4.55, *p* = .000. The study received approval from the Kenyatta University Ethics Review Board and all participants gave an informed consent for participation in the study.

### 3.3 Analysis

#### 3.3.1 Measurement invariance

We evaluated measurement invariance by country identification in two student samples. Fit statistics for all invariance tests (see Table 8) were interpreted in accordance with recommendations proposed by Hu and Bentler (1999): CFI values >.95 indicate acceptable model fit; RMSEA values below .06 suggest good model fit; and SRMR values <.08 indicate acceptable model fit. The metric invariance model tested whether the structure of factor loadings was equal across the two groups. Invariance is supported when model fit is not notably poorer than the configural model (Putnick and Bornstein, 2016). Finally, the scalar invariance model tests for group differences in item intercepts and is examined by comparing model fit to the metric model. Measurement invariance literature suggests that when comparing the metric model to the configural model and the scalar model to the metric model, the chi-square difference test-statistic may suggest non-invariance as a result of small changes in model fit since it is overly sensitive to large sample size (Chen, 2007; Kline 2015; Putnick and Boornstein, 2016). Thus, in addition to chi-square difference tests, we examined the following fit statistics using cutoffs from Chen (2007) to evaluate non-invariance: a change in CFI ≤ −.005 in addition to a change of ≥.010 in RMSEA or a change of ≥.025 in SRMR indicates metric non-invariance, and a CFI change ≤ −.005 in addition to a change of ≥.010 in RMSEA or a change of ≥.005 in SRMR indicates scalar non-invariance.

**Table 8:** Measurement invariance for ATFMUS across Ghanaian and Kenyan samples.

Model	χ^2^ (df)	CFI	RMSEA [90% CI]	SRMR	Model Comp	Δχ^2^ (Δdf)	ΔCFI	ΔRMSEA	ΔSRMR	Invariant?
*Country (*Kenya, *n* = 199; Ghana, *n* = 242)										
M1: Configural	40.022∗∗ (10)	.898	.083 [.057, .110]	.043		--	--	--	--	
M2: Metric	44.606∗∗(14)	.896	.071 [.048, .094]	.049	M1	4.584 (4)	−.002	−.012	.006	Yes
M3: Scalar	46.495∗∗ (18)	.903	.060 [.039, .082]	.050	M2	1.889 (4)	.007	−.011	.001	Yes
M4: Residual	48.080∗∗(23)	.915	.050 [.030, .070]	.050	M3	1.585 (5)	.012	−.010	.000	Yes

*Note. N* = 557. M = Model. ∗∗*p* ≤ .01.

We evaluated configural invariance following the recommendations proposed by Hu and Bentler (1999) for good model fit as indicated by: CFI values >.95; RMSEA values below .06; and SRMR values <.08. The indices found in our study of CFI = .90; RMSEA = .08; and SRMR = .04 approximated a good model fit. We therefore proceeded to evaluate metric invariance by comparing the configural model to the metric model using a chi-square difference (Δ χ^2^) test. The test was non-significant Δ χ^2^ = 4.584, *df* = 4, *p* = .333. In addition, changes in the alternative fit indices (ΔAFIs) were considered (i.e. Δ*CFI* = −.002, Δ*RMSEA* = −.012 and Δ*SRMR* = .006) and they all suggested that metric invariance was established. This finding implied that the model did not change significantly after constraining the factor loadings to be equal for the two groups. Thus the meaning of the ATFMUS items can be said to be comparable across participants from Ghana and Kenya. We thus moved to the next step of establishing scalar invariance by comparing the scalar model to the metric model. The Δχ^2^ test was not significant Δ χ^2^ = 1.889, *df* = 4, *p* = .756 suggesting scalar invariance. We further examined the ΔAFIs (i.e. Δ*CFI* = .007, Δ*RMSEA* = −.011 and Δ*SRMR* = .001) which provided further evidence of scalar invariance as per the traditional criteria of -.01 for ΔCFI and .01 for ΔRMSEA (Chen, 2007; Putnick and Bornstein, 2016). In the next step we checked for strict invariance by constraining the residuals to be equal for the Kenyan and Ghanaian participants. The chi-square difference test was not significant Δ χ^2^ = 1.585, *df* = 5, *p* = .903 suggesting that ATFMUS indicates strict invariance across participants from Ghana and Kenya. The ΔAFIs (i.e. Δ*CFI* = .012, Δ*RMSEA* = −.010 and Δ*SRMR* = .000) added evidence of residual invariance as per the traditional criteria of -.01 for ΔCFI and .01 for ΔRMSEA (Chen, 2007; Putnick and Bornstein, 2016).

Having established measurement invariance, we proceeded to test for mean differences in the ATFMUS items across groups using independent samples t-tests as presented in Table 9.

**Table 9:** Descriptive statistics and mean differences for the ATFMUS items for the Ghanaian and Kenyan samples.

	Country	M	SD	Sk	Kur	t	p
The idea of using face masks is not appealing	Ghana	2.73	1.41	.26	−1.22	.370	.711
	Kenya	2.68	1.50	.28	−1.37		
I would avoid using a face mask if possible	Ghana	2.90	1.48	.11	−1.38	−.227	.821
	Kenya	2.93	1.66	.08	−1.63		
I just don't like the idea of using face masks	Ghana	3.02	1.41	−.05	−1.27	.752	.453
	Kenya	2.91	1.62	.04	−1.59		
I only wear a face mask when I know I am likely to be punished for not wearing one	Ghana	3.48	1.48	−.41	−1.30	.912	.362
	Kenya	3.34	1.63	−.34	−1.52		
Face masks are unhygienic	Ghana	3.67	1.50	−.70	−1.01	−.191	.849
	Kenya	3.70	1.51	−.71	−.98		

*Note. N* = 557. Ghana *n* = 242, Kenya *n* = 199. *Sk* = Skewness, *Kur* = Kurtosis.

As presented in Table 9, there were no significant differences in the items mean scores for participants from Ghana and Kenya.

## 4 Discussion

Despite availability of studies on attitudes towards mask use, the psychometric properties of the available instruments are apparently not adequately established which may limit their usefulness in studying attitudes towards PFMU across contexts. We set out to develop the attitudes towards face mask use scale (ATFMUS) and validated it using college and high school samples drawn from Kenya and Ghana. To the best of our knowledge, this was the first study to specifically develop, evaluate and confirm the psychometric properties of a brief scale to measure attitudes towards face mask use in an Africa context. We developed the AFTMUS in three phases: item generation, scale development, and scale evaluation. Phase one and two were addressed in study 1 while phase three was addressed in studies 2 and 3.

In study 1, we developed the ATFMUS grounded on the rational-theoretical approach (Hubley and Zumbo, 2013) by reviewing relevant literature (e.g. Rieger 2020; Sikakulya et al., 2021; Taylor and Asmundson, 2021) and consulting psychology experts on attitudes towards use of personal health protective equipment. We came up with a pool of 19 items focusing on: (1) perceptions on usefulness and effectiveness of face masks, (2) behaviours related to facemask use in the context of COVID-19 prevention, (3) opinions about physical and interactional side effects of facemasks. The three categories are well aligned to the model proposed by Esmaeilzadeh (2022). We used the criteria reported in scale development literature (Boateng et al., 2018; Jin et al., 2018; Lester et al., 2014) to conduct a preliminary item analysis for the ATFMUS. Consequently, four items that had kurtosis and skewness values higher than |2| and five items that had item-total correlations below 0.3 were removed. All the dropped items had means above the cut off value of 4.2. An exploratory factor analysis suggested a 2-factor solution while and a parallel analysis (Horn, 1965) supported a one-factor solution for the remaining 10 items of the ATFMUS. The study yielded robust evidence for internal consistency reliability with the one-factor model having α = .80, while factors one and two had α = .77 and α = .73 respectively in the two-factor model. This provided evidence that the ATFMUS has sufficient levels of reliability in its use to measure attitudes towards face mask use.

In Study 2, we performed a CFA and the 10-item model did not quite meet the desired values for acceptable fit (Chen, 2007). Through fit checks on standardized residual covariances, five items were iteratively dropped yielding a best fit model. Thus, consistent with our hypothesis, the ATFMUS was better conceptualized as a 5-item one factor scale rather than a two-factor scale. The items retained in ATFMUS all focused on negative aspects of facemask use and therefore the scale may function well in capturing negative attitudes towards face mask use. Reliability analysis for the final 5-item ATFMUS yielded an alpha of .71 meeting the criteria for a reliable scale as per the good practice guidelines in scale development literature that specify an alpha of .70 or higher as acceptable (Boateng et al., 2018; Haladyna, 2016). Measurement invariance analysis gave initial evidence that the ATFMUS scores have consistent meanings across sex, age, and level of education. The scale also yielded moderate effect sizes in known group comparisons providing initial evidence that it was sensitive to whether participants always wore masks in public or not; whether they believed there is Corona Virus or not; and whether they agreed that face masks can prevent Corona Virus.

The study also provided robust evidence of convergent and discriminant validity of the ATFMUS. Consistent with our hypotheses, ATFMUS had weak-to-moderate correlations with personality traits (consciousness and agreeableness); exaggerating of positive qualities; obsession with Covid-19, and Corona Anxiety. On the other hand, ATFMUS had negative correlations with minimizing negative qualities; and negative affect. These results are consistent with those reported in other studies on attitudes towards face mask use (e.g. Duong et al., 2021; Larebo and Abame, 2021; Taylor et al., 2020; Wismans et al., 2022). Considering the novelty of the ATFMUS, further studies are needed to confirm these interesting results.

In study 3, ATFMUS had equivalent meaning across participants in Ghana and Kenya. In our study, the CFI increased in scalar and residual models while the RMSEA decreased implying that these models had a better fit than the configural and metric models. That ATFMUS had improvement in fit of its more restrictive models is quite a convincing evidence of its fairness across the countries. In addition, the item means did not differ across the two countries. This implies a great potential for use and research across cultural and geographical settings. Taken together, the findings support the psychometric properties of the ATFMUS as a brief and easy-to-use instrument that assesses attitudes towards face mask use across participants of different age, level of education, and cultural backgrounds.

### 4.1 Strengths, limitations, and future research directions

The key strengths of this study were that we involved relatively large samples to develop and validate the ATFMUS. In addition, we tested for the scale's invariance across two countries. To the best of our knowledge, this was the first study to specifically develop, evaluate and confirm the psychometric properties of a brief scale to measure attitudes towards face mask use in an African context. Despite its contributions, this study had some limitations that are worthy noting. First, the items included in the ATFMUS may not assess all face-mask use related attitudes. Future research may investigate the face mask use attitudes that were not investigated in the present study. Second, we did not test for the test-retest reliability and the responsiveness of the ATFMUS since we used a cross-sectional analysis. Third, we mainly used student samples from public institutions. We encourage future studies to evaluate measurement invariance in both community and student samples since the two are the mostly studied populations in psychological assessment research (Boateng et al., 2018). Third, we only used evidence from Kenya and Ghana to establish cross-country applicability of ATFMUS. We recommend that future studies could extend its international use by involving more countries in cross-cultural and cross-country invariance analysis. Such work could involve larger samples per country than we used in this study. Fourth, we did not investigate how ATFMUS correlates with indicators of the COVID-19 disregard “syndrome” (Taylor et al., 2020) such as feelings of personal invulnerability, risk compensation behavior, perception of the COVID-19 as being overrated and disregard of prevention protocols. Future studies could explore such.

## 4.2 Conclusion

In this study, we developed the ATFMUS and evaluated its factor structure, measurement invariance, internal construct validity, convergent validity, known-group validity and internal consistency among Ghanaian and Kenyan participants. Our findings suggest that the ATFMUS is a reliable and valid scale for assessing attitudes towards face mask use. The results reveal that the 5-item version of the ATFMUS is a brief and easy-to-use instrument that assesses attitudes towards face mask use across participants of different age, level of education, and cultural backgrounds. The scale is also sensitive to participants’ actual use of face masks, and their beliefs about COVID-19 and efficacy of the facemasks. Thus ATFMUS is a worthwhile addition to existing scales for measuring attitudes towards face mask use and this study may serve as a foundation for further validation of the scale.

## Declarations

### Author contribution statement

Anthony Ireri; Cecilia Nyambura Mwangi; Vera Arhin: Conceived and designed the experiments; Performed the experiments; Analyzed and interpreted the data; Contributed reagents, materials, analysis tools or data; Wrote the paper.

Martha Akoth; Stephen Mugo; Ruth Ncororo Munanu: Conceived and designed the experiments; Contributed reagents, materials, analysis tools or data; Wrote the paper.

### Funding statement

This research did not receive any specific grant from funding agencies in the public, commercial, or not-for-profit sectors.

### Data availability statement

Data will be made available on request.

### Declaration of interest’s statement

The authors declare no conflict of interest.

### Additional information

Supplementary content related to this article has been published online at [URL].

## Appendix A Supplementary data

ATFMUS STUDY QUESTIONNAIRE ATFMUS STUDY QUESTIONNAIRE
