# Supplementary material for: Development and initial validation of the attitudes toward face mask use scale (ATFMUS)
Source: Heliyon. 2022 Dec 19;8(12):e12349. doi: 10.1016/j.heliyon.2022.e12349 (PMC9813729; doi:10.1016/j.heliyon.2022.e12349)
Supplement: ATFMUS STUDY QUESTIONNAIRE [file mmc1.docx]

Dear participant,

**General Instructions:**

This questionnaire collects data as part of a larger study on attitudes and behaviour during the Coronavirus (Covid-19) pandemic. The questionnaire has two pages and should take you at most 30 minutes to complete. Although some of the statements may seem similar to one another, they differ in important ways. For each item, please indicate your answer by placing a tick **(√)** on the number that corresponds to your answer on the given scale. Please be as honest and accurate as you can. Try not to let your response to one statement influence your responses to other statements. There are no "correct" or "incorrect" answers so respond according to your own feelings.

Your participation is purely voluntary. Note that all information collected in this study will be used for research purposes only and will be held in strict confidentiality. Should you have any queries, kindly contact the lead researcher on:….

………..

1. Demographic information: Please indicate your: (i) Gender----------- (ii) Level of Education-------- (e.g. Primary, Secondary, University, College) (iii) Age--------Years (iv) The Country where you are now…………………..
2. Tick **(√)** *Yes* or *No* for each of the following statements:

| Statement | **Yes** | **No** |
| --- | --- | --- |
| Do you always wear a face mask when you are in public? |  |  |
| Do you believe there is coronavirus? |  |  |
| Can a face mask prevent coronavirus? |  |  |

1. (ATFMUS) The following statements focus on your feelings about USE OF FACE MASKS. Indicate how you agree with each, answer using the scale: 1= Strongly Disagree (SD); 2= Disagree a little (D); 3= Neither agree nor disagree (N), 4= Agree a little (A), and 5= Strongly Agree (SA)

|  | Statement | SD | D | N | A | SA |
| --- | --- | --- | --- | --- | --- | --- |
|  | Face masks are uncomfortable | 1 | 2 | 3 | 4 | 5 |
|  | The idea of using face masks is not appealing | 1 | 2 | 3 | 4 | 5 |
|  | Proper use of face masks can enhance one’s safety from the coronavirus | 1 | 2 | 3 | 4 | 5 |
|  | I intend to use always use face masks as long as there is the coronavirus | 1 | 2 | 3 | 4 | 5 |
|  | I would be comfortable suggesting to a friend to use a face mask | 1 | 2 | 3 | 4 | 5 |
|  | I would avoid using a face mask if possible | 1 | 2 | 3 | 4 | 5 |
|  | I just don’t like the idea of using face masks | 1 | 2 | 3 | 4 | 5 |
|  | People who use face masks show concern and responsibility to those around them | 1 | 2 | 3 | 4 | 5 |
|  | Using face masks is unfashionable | 1 | 2 | 3 | 4 | 5 |
|  | A face mask is the best way to protect myself from the coronavirus | 1 | 2 | 3 | 4 | 5 |
|  | It is polite to wear a face mask in public | 1 | 2 | 3 | 4 | 5 |
|  | Wearing a face mask may make friends think that you have the coronavirus | 1 | 2 | 3 | 4 | 5 |
|  | I only wear a face mask when I know I am likely to be punished for not wearing one | 1 | 2 | 3 | 4 | 5 |
|  | Face masks are unhygienic | 1 | 2 | 3 | 4 | 5 |
|  | People are likely to stare at me more when I wear a face mask | 1 | 2 | 3 | 4 | 5 |
|  | Sometimes I don’t wear a face mask so that I can be like the people near me | 1 | 2 | 3 | 4 | 5 |
|  | A face mask makes breathing difficult | 1 | 2 | 3 | 4 | 5 |
|  | Wearing face masks may not change anything because there is no coronavirus | 1 | 2 | 3 | 4 | 5 |
|  | As long as I socially distance, I don’t need a face mask | 1 | 2 | 3 | 4 | 5 |

*THANK YOU FOR YOUR PARTICIPATION*
